# In-Situ Gold–Ceria Nanoparticles: Superior Optical Fluorescence Quenching Sensor for Dissolved Oxygen

**DOI:** 10.3390/nano10020314

**Published:** 2020-02-12

**Authors:** Nader Shehata, Ishac Kandas, Effat Samir

**Affiliations:** 1 Center of Smart Nanotechnology and Photonics (CSNP), SmartCI Research Center, Alexandria University, Alexandria 21544, Egypt; ishac@vt.edu; 2 Department of Engineering Mathematics and Physics, Faculty of Engineering, Alexandria University, Alexandria 21544, Egypt; 3 USTAR Bio-innovation center, Utah State University, Logan, UT 84341, USA; 4 Kuwait College of Science and Technology, Doha Area, 7th Ring Road, Safat 13133, Kuwait; 5 The Bradley Department of Electrical and Computer Engineering, Virginia Tech, Blacksburg, VA 24061, USA; 6 Department of Electrical Engineering, Old Dominion University, Norfolk, VA 23508, USA; effat_samir@mena.vt.edu

**Keywords:** ceria, dissolved oxygen, gold nanoparticles, sensing, fluorescence quenching

## Abstract

Cerium oxide (ceria) nanoparticles (NPs) have been proved to be an efficient optical fluorescent material through generating visible emission (~530 nm) under violet excitation. This feature allowed ceria NPs to be used as an optical sensor via the fluorescence quenching Technique. In this paper, the impact of in-situ embedded gold nanoparticles (Au NPs) inside ceria nanoparticles was studied. Then, gold–ceria NPs were used for sensing dissolved oxygen (DO) in aqueous media. It was observed that both fluorescence intensity and lifetime were changed due to increased concentration of DO. Added gold was found to enhance the sensitivity of ceria to DO quencher detection. This enhancement was due to optical coupling between the fluorescence emission spectrum of ceria with the surface plasmonic resonance of gold nanoparticles. In addition, gold caused the decrease of ceria nanoparticles’ bandgap, which indicates the formation of more oxygen vacancies inside the non-stoichiometric crystalline structure of ceria. The Stern–Volmer constant, which indicates the sensitivity of optical sensing material, of ceria–gold NPs with added DO was found to be 893.7 M^−1^, compared to 184.6 M^−1^ to in case of ceria nanoparticles only, which indicates a superior optical sensitivity to DO compared to other optical sensing materials used in the literature to detect DO. Moreover, the fluorescence lifetime was found to be changed according to the variation of added DO concentration. The optically-sensitivity-enhanced ceria nanoparticles due to embedded gold nanoparticles can be a promising sensing host for dissolved oxygen in a wide variety of applications including biomedicine and water quality monitoring.

## 1. Introduction

Fluorescence quenching mechanisms are widely used for optical sensing, depending on fluorescent nanostructures. An efficient fluorescent nanostructure is needed to obtain an acceptable level of emission so that it can be easily detected. Different fluorescent nanostructures can be used, such as different dimensional silicon nanomaterial for sensing chemical and biological species, lead for sensing graphine oxide, silicon nanowires for sensing Cu, silica-coated QDs for the detection of dopamine and glutathione in physiologically relevant concentrations [1,2,3,4]. Cerium oxide (ceria) is considered as one of the most efficient fluorescent nanostructures due to its visible emission under ultraviolet excitation, in addition to its reduction–oxidation properties [5,6,7,8]. Non-stoichiometric ceria nanoparticles are found to have a ratio of tri-valent cerium ions, which forms a trap state inside the energy level structure of ceria which corresponds to visible emission (centralized at around 520 nm) [9]. In addition, the formed tri-valent cerium ions are associated with the formation of oxygen vacancies which can adsorb some quenchers such as dissolved oxygen (DO) and radicals [10,11]. This adsorption can lead to fluorescence intensity quenching the visible emission of ceria nanoparticles. However, the optical sensitivity of ceria nanoparticles is found to be quite low compared to some more expensive and famous optical sensing hosts such as ruthenium [12]. Thud, there is still a need to raise the optical sensitivity of ceria nanoparticles to quench some elements such as dissolved oxygen, which is important for water quality monitoring, fishery, and biomedical applications [13,14,15,16]. Therefore, our idea in this work was to embed gold nanoparticles within ceria nanoparticles via in-situ synthesis technique and use the formed nanocomposite as an optical sensing host for DO. Plasmonic nanostructures, such as gold nanoparticles (Au NPs), can enhance the emission intensity of fluorescence nanostructures. Metal type, nanoparticle size, and distance between the particle and fluorescence nanostructure are important factors to be considered within plasmonic-based-sensor [17]. An Au NPs-based sensor has been developed for detecting Hg(II) ions in aqueous solution [18]. An enhancement of the fluorescence intensity by more than a factor of 50 was recorded for indocyanine green (ICG) next to a nanoparticle [19]. A series of plasmonic nano slits were designed and used for immobilization of Carbon nanodots (CNDs), so a fluorescence enhancement of CNDs was noticed [20]. Au NPs induced metal-enhanced fluorescence (MEF) for a modified bipyridine-based construct 4-(pyridine-2-yl)-3H-pyrrolo[2,3-c]quinoline (PPQ) bounded with biologically important Zn^2+^ [21]. A fluorescence quenching mechanism is exploited for sensing the concentration of a substance. From the literature, we can embed gold nanoparticles within other nanostructures, such ceria nanoparticles, depending on the optical sensitivity contribution of the surface plasmonic waves of the added gold nanoparticles. There are previous research studies that used ceria nanoparticles or lanthanide-doped-ceria nanoparticles as optical nanosensors for dissolved oxygen [11], but the addition of plasmonic nanostructures within ceria is still lacking.

In this work, we intended to use gold–ceria NPs for sensing dissolved oxygen (DO) with different concentrations. Then, our idea was the careful selection of plasmonic NPs’ resonance wavelength to be overlapped with the peak wavelength of the fluorescence emission of ceria nanoparticles, and consequently, to ensure the enhancement of fluorescence emission intensity along with the corresponding optical detection’s sensitivity. The detection mechanism of variable concentrations is the optical fluorescence quenching of both intensity and lifetime. Quenching means the decrease of the emission intensity due to increased concentration of quencher; which is DO in this presented work. The corresponding Stern–Volmer constant, which indicates sensitivity to detect the quenchers [16], would be calculated through the relative change of fluorescence emission intensity in both the absence and presence of the quencher. Another indicator to be considered is the change in lifetime due to adding the DO. Lifetime is the time spent in the excitation state before returning back to the ground state, which can be affected by the quencher (DO) and can be another measure of its concentration. By the end of the paper, we will have presented a simple design of a sensor prototype using our synthesized ceria nanoparticles as a sensing material to DO in aqueous media.

## 2. Materials and Methods

For the optical characterizations and sensing experiment, a Simple fluorescence spectroscopy setup was used to measure the intensity of the fluorescence emission. The system consists of a light emitting diode (LED) for excitation at wavelength of ~430 nm. The sample was put in a fluorescence quartz cuvette and was exposed to the light coming from the LED. The emitted light was collected by passing through a monochromator (Cornerstone 130 monochromator, Newport, Irvine, CA, USA). The output intensity from the monochromator was amplified using photomultiplier tube (PMT) (Newport 77360, Irvine, CA, USA). Finally, a power meter (1918R) was used to record the intensity for each wavelength. For lifetime measurement, the same setup was used with a small modification. An optical chopper (MC2000B, Thorlab, Newton, NJ, USA) with a chopping frequency up to 10 kHz was added in front of the excitation LED. A total integration time of PMT of 22 ns was used while the detection method was the same. Then, DO was added to the solution at different concentrations.

For sensing the material’s synthesis, Ceria NPs were synthesized using a chemical precipitation technique for both relatively low-cost precursors and simplicity [22]. In detail, 0.5 g of cerium chloride (Sigma Aldrich, St. Louis, MO, USA) was added in 40 mL of distilled water. Then, the solution was stirred for two hours at a rotation rate of 500 rpm and a temperature of 50 °C for two hours at a stirring speed 500 rpm. In the beginning of stirring, 1.6 mL of ammonium hydroxide (ammonia) was added as a catalyst. After two hours of warm stirring, the solution was stirred at same rotation speed at room temperature overnight. This long stirring process helps to break most of formed ceria nanorods into NPs, as shown in Figure S1. Moreover, the first warming stage helped in converting cerium hydroxide into non-stoichiometric ceria NPs. The diameter of the used gold nanoparticles (Au NPs) (Sigma Aldrich) was selected as 20 nm to have a plasmonic resonance wavelength overlapped with a fluorescence emission wavelength of 530 nm. Au NPs were added in situ within initial precursors’ addition stage of the synthesis process to ensure a better surround of the gold nanoparticles within the ceria nanoparticles structure.

The absorbance dispersion was measured using UV–Vis spectroscopy (Shimadzu 2600, Tokyo, Japan). Images of gold-ceria NPs were obtained using a transmission electron microscope (TEM) (JEOL, Tokyo, Japan) with an accelerating potential of 80 kV.

## 3. Results and Discussion

**Figure 1:**
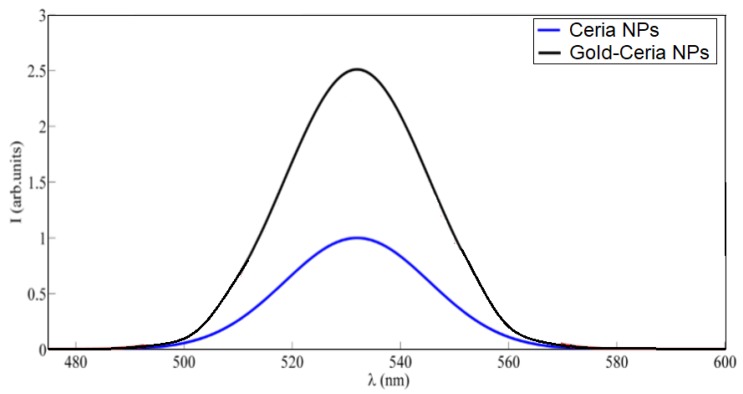
Emission intensity spectrum of both ceria nanoparticles (NPs) only and gold–ceria NPs under 430 nm excitation.

**Figure 2:**
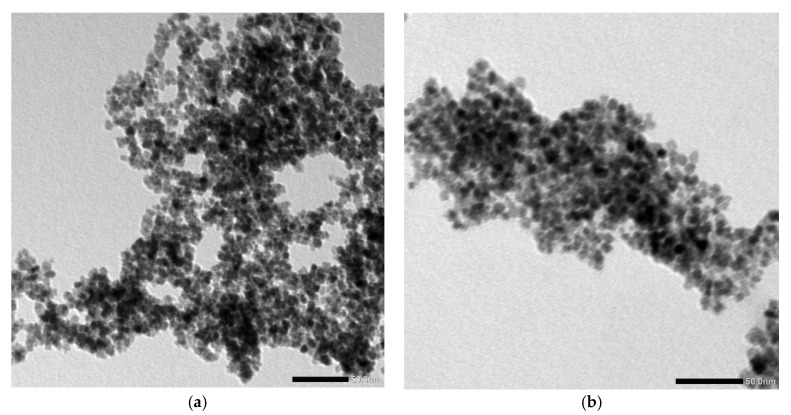
TEM image of (**a**) ceria nanoparticles and (**b**) gold–ceria nanoparticles.

In Figure 1, the fluorescence emission of gold–ceria NPs is shown with and without added in-situ gold nanoparticles, under violet excitation of 430 nm. The generated fluorescence emission of ceria corresponds to 5d-4f molecular transition [9]. It can be seen that plasmonic gold nanoparticles increase the fluorescence intensity emission of ceria nanoparticles. This possibly resulted from the overlap between fluorescence emission spectrum of ceria, of peak close to 530 nm, with the selected gold plasmonic resonance wavelength of 530 nm. Figure 2 shows a TEM image of synthesized gold-ceria nanoparticles, with a mean diameter of gold nanoparticles of 20 nm and a slightly smaller diameter for ceria nanoparticles.

**Figure 3:**
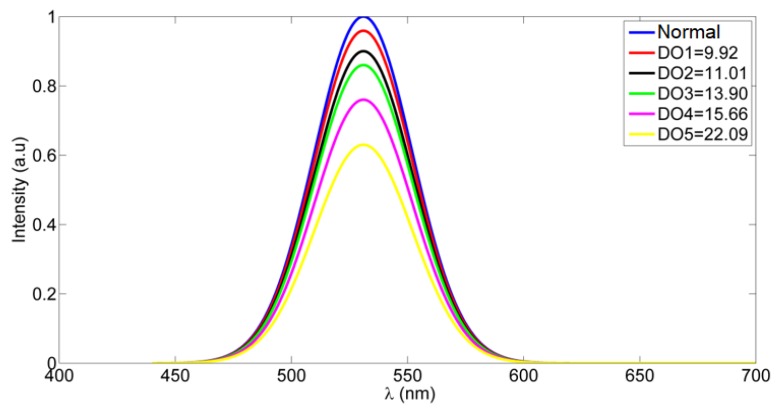
Emission intensity quenching of in-situ adding of Au NPs at different concentrations of dissolved oxygen (DO). The word “Normal” means gold-ceria nanoparticles in normal distilled water with no further added DO concentration, where the measured DO was 7.8 mg/L. All mentioned concentrations of DO in the legend are in mg/L.

**Figure 4:**
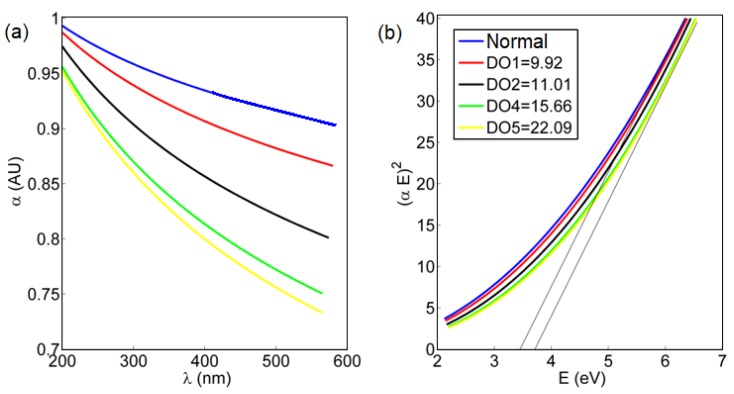
(**a**) Absorbance dispersion; and (**b**) bandgap calculations for gold-ceria NPs with different added DO concentrations. Normal means the default DO concentration in distilled water with no further added oxygen, which was measured to be 7.8 mg/L. All mentioned DO concentrations are in units of mg/L. Legend box is common for both Figure 4a,b.

Now, the fluorescence emission of gold-ceria nanoparticles is shown through added DO at different concentrations, as shown in Figure 3. The fluorescence intensity was quenched due to the increased DO concentration. This quenching is due to the adsorption of dissolved oxygen within the formed oxygen vacancies inside the crystalline structure of the ceria nanoparticles. To detect the mechanism of quenching, the absorbance dispersion was measured for gold–ceria nanoparticles at different added DO concentrations, as shown in Figure 4a. Then, the corresponding bandgap of ceria nanoparticles was calculated through the following equation [23]

*αE* = A(*E* − *E*_*g*_)^1/2^ (1)

where *α* is the optical absorbance, A is a material-dependent constant related to effective masses of electrons and holes, *E* is the absorbed photon energy, and *Eg* is the allowed direct bandgap. From both Figure 4a,b, the blue-shift in both absorbance and bandgap is observed due to an incremental concentration of added dissolved oxygen. This proves the dominant static adsorption nature of gold-ceria NPs to dissolved oxygen due to the increased formation of charged oxygen vacancies associated with the more formed tri-valent cerium ions.

**Figure 5:**
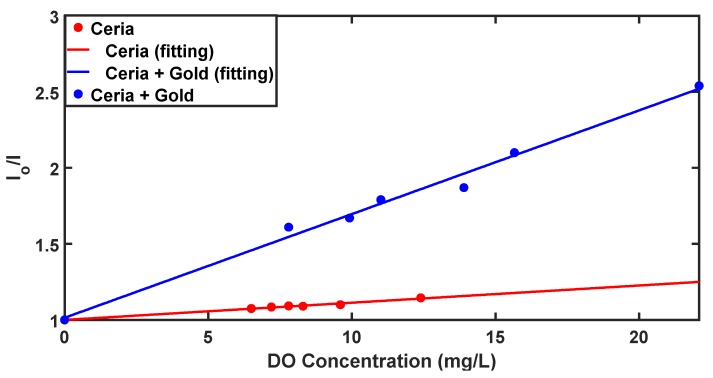
Stern–Volmer analysis of DO optical detection using ceria NPs and gold-ceria NPs.

Depending on the results from Figure 3, the relative intensity versus the concentration of added DO is plotted in Figure 5. The relative intensity is calculated using the Stern–Volmer equation [11]:

(2) $$\frac{I_{o}}{I}=1+K_{SV}\left[Q\right]$$

where *I_o_* is the peak intensity in the absence of quencher of DO, *I* is the peak intensity in the presence of DO, *K_SV_* is the Stern–Volmer quenching constant and *Q* is DO concentration. From Figure 5, the relation is almost linear and K_SV_ is calculated to be 893.7 M^−1^ for gold-ceria NPs with added DO, compared to 184.6 M^−1^ for ceria NPs only, which means a superior increment in the optical sensitivity of the material by 4.85 times due to embedded gold nanoparticles. However, the linearity is slightly less due to the added gold nanoparticles with a quite larger deviation in readings, compared to the pure ceria nanoparticles case. In addition, our newly gold–ceria nanoparticles were found to have higher *K_SV_*, and consequently, the sensing sensitivity to DO was more quenched compared to other sensing materials used in literature which had a range of *K_SV_* from less than 1 M^−1^ to 115 M^−1^ [24,25,26].

**Figure 6:**
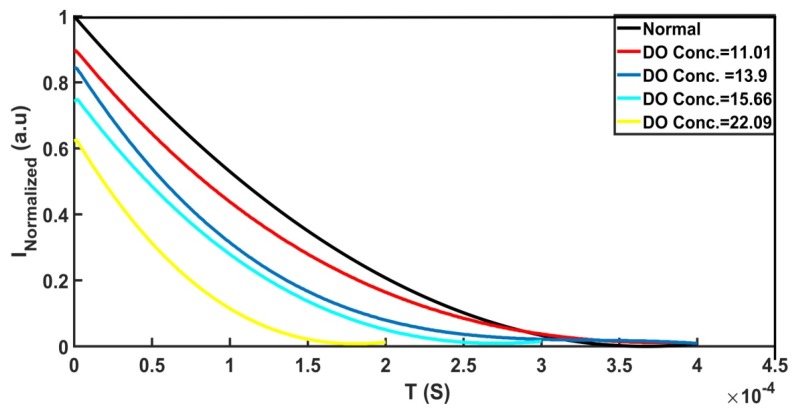
Intensity decay versus time of gold–ceria nanoparticles at different DO concentrations (in mg/L).

**Figure 7:**
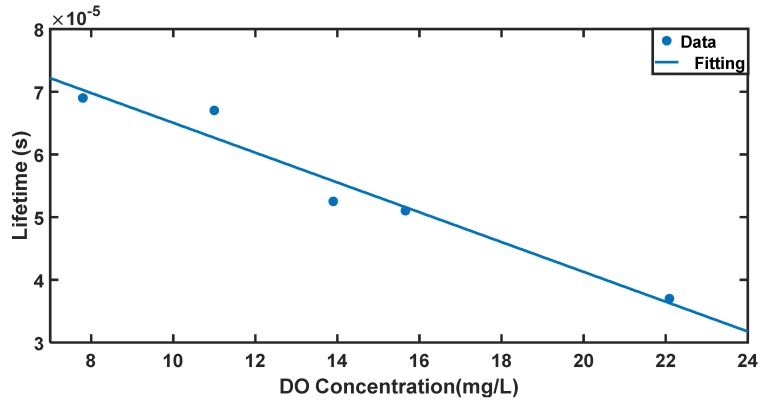
Linear relation between fluorescence lifetime and DO concentration at DO range from 7.8 to 22 mg/L.

Another sensing parameter is the optical lifetime of gold-ceria nanoparticles with variable DO concentrations, as shown in Figure 6. It can be noticed that the fluorescence lifetime was reduced due to the increased concentration of the DO quencher from 0.069 ms up to 0.037 ms for the DO concentration range from 7.8 ppm to 22.09 ppm. The lifetime variation with added different DO quencher concentrations is shown in Figure 7. The linear fitting of lifetime values versus DO concentrations shows a sensitivity of −2.3 μs/ppm for the aforementioned range of DO concentration. That can help to offer another tool for optical measurement of DO concentrations in aqueous media based on fluorescence lifetime.

**Figure 8:**
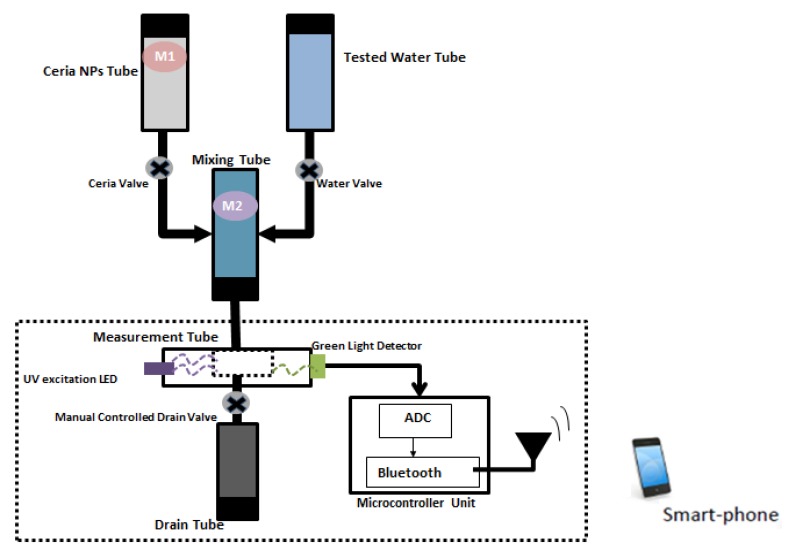
Schematic diagram of optical sensor unit along with communication unit.

As an analogy to real life application for detecting different water quality conditions according to DO concentrations, we implemented our sensing host to detect the dissolved oxygen in water. Then, the normal water detection of DO around 8 ppm was considered the reference signal in the microcontroller unit, and any change in DO concentration can be comparable to the reference signal. Figure 8 shows a schematic diagram of a basic compact automatic sensor. The designed prototype mainly depends on using a commercial optical detector to sense the variation on quenchers into water and send it using the flexible paper-based antenna to a closed communication node. The proposed prototype includes some mechanical components such as motors and solenoid valves, which are fully controlled by a compact microcontroller unit (Arduino). The main aspects of the designed optical sensor can be summarized into the following points:

- Four tubes to store the main elements (sample of tested water, stock of ceria NPs solution, Mixing tube, optical sensor tube).
  - Mixing tube: It contains a specific amount of ceria NPs, and it mixes such amount of ceria with a sample of the required tested water.
  - Optical sensor tube: It contains the main optical sensing equipment, which are cuvette, UV LED, and green light detector.
- Pumping system: It includes solenoid valves to pull certain quantity form ceria NPs solution and tested water into mixing tube. Additionally, another system to pump that mixture in the optical sensor tube to drain tube and start new testing.
- Mixing/shaking system: It ensures a constant concentration of ceria into solvable solution tube, and prevents ceria NPs precipitation inside the tube.
- Analog-to-Digital (ADC) system: ADC system is used for mapping the analog reading signals form photo detector element into digital signals.
- Processing system: It is a simple compact-size microcontroller system (arduino) connected to a Bluetooth module, as shown in Figure S2. It is mainly used to controller the motor and solenoid valves and converts the analog reading signals into digital reading signals. Additionally, it sends a live status of sensor to cellphone through the attached Bluetooth module with paper-based antenna. Such a displayed reading is used to decide the quality of the tested water based on reference calculations.
- Power supply system: Most system elements are powered through a DC power supply.

The main process of the designed prototype relies on optical sensor components, which are a UV LED, cuvette, and green optical sensor. Such elements are integrated on a small isolated tube. This tube is connected to another two tubes (ceria NPs and the tested water) to perform measurements and analysis. The ceria NPs and the tested water are mixed together into the mixing tube and feed this mixture to the optical sensor tube. On the other hand, another two valves are used to feed this mixing tube. The first valve is used to pull ceria NPs that exists in another third tube with a shaking system to the mixing tube, while the other valve is used to pull the required tested water to the same mixing tube. After all measurements have been performed, the user has to press the push button to drain the tested sample and start a new test with fresh samples.

## 4. Conclusions

In this work, gold-ceria nanoparticles were used as an optical sensing material for dissolved oxygen (DO). Gold nanoparticles (Au NPs) were embedded in situ within ceria nanoparticles to enhance the optical fluorescence intensity due to overlap between the resonance wavelength of Au NPs and the emission of ceria NPs. The fluorescence emission intensity of gold-ceria NPs was found to be quenched according to the increment of DO concentration. Stern–Volmer constant is was to be 893.7 M^−1^ with static quenching nature due to the variation of absorbance and bandgap at different DO concentrations. This sensitivity was superior compared to pure ceria nanoparticles or other sensing materials studied in the literature. Moreover, the optical fluorescence lifetime was found to be changed according to the DO quencher. Finally, a prototype of the used implemented ceria nanoparticles as a sensor unit was presented. This work is useful for DO detection in biomedicine and water quality monitoring.

## Supplementary Materials

The following are available online at https://www.mdpi.com/2079-4991/10/2/314/s1, Figure S1: Formation of mixed ceria nanorods and nanoparticles with relatively short stirring synthesis; Figure S2: Bluetooth module connection with Arduino.
