# Supplementary material for: In-Situ Gold–Ceria Nanoparticles: Superior Optical Fluorescence Quenching Sensor for Dissolved Oxygen
Source: Nanomaterials (Basel). 2020 Feb 12;10(2):314. doi: 10.3390/nano10020314 (PMC7075203; doi:10.3390/nano10020314)
Supplement: Supplementary file 1 [file nanomaterials-10-00314-s001.pdf]

nanomaterials

Supplementary Materials: In-Situ Gold–Ceria Nanoparticles: Superior Optical Fluorescence Quenching Sensor for Dissolved Oxygen

Nader Shehata [1,2,3,4,5,*], Ishac Kandas [1,2,4] and Effat Samir [6]

[1] Center of Smart Nanotechnology and Photonics (CSNP), SmartCI Research Center, Alexandria University, Alexandria 21544, Egypt; ishac@vt.edu
[2] Department of Engineering Mathematics and Physics, Faculty of Engineering, Alexandria University, Alexandria 21544, Egypt
[3] USTAR Bio-innovation center, Utah State University, Logan, UT 84341, USA
[4] Kuwait College of Science and Technology, Doha Area, 7th Ring Road, Safat 13133, Kuwait
[5] The Bradley Department of Electrical and Computer Engineering, Virginia Tech, Blacksburg, VA 24061, USA
[6] Department of Electrical Engineering, Old Dominion University, Norfolk, VA 23508, USA; effat_samir@mena.vt.edu
* Correspondence: nader83@vt.edu; Tel.: +20-109-116-5300

Figure S1 shows the formation of mixed ceria nanorods and nanoparticles along with relatively low stirring period. That would support the need of long stirring period to guarantee the complete formation of nanoparticles. Figure S2 shows the connection between Arduino microcontroller with the Bluetooth module.

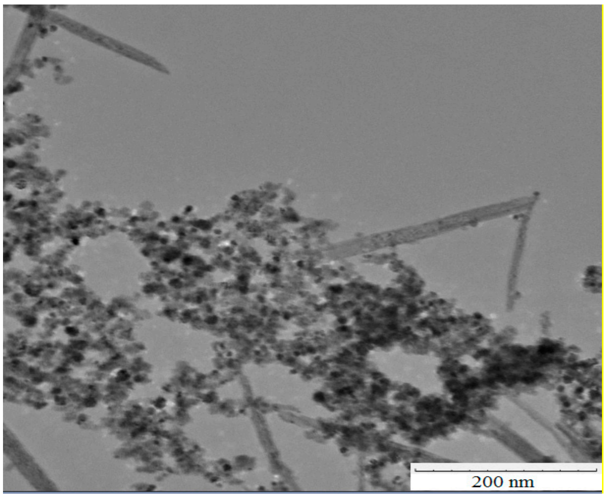

Figure S1. Formation of mixed ceria nanorods and nanoparticles with relatively short stirring synthesis.

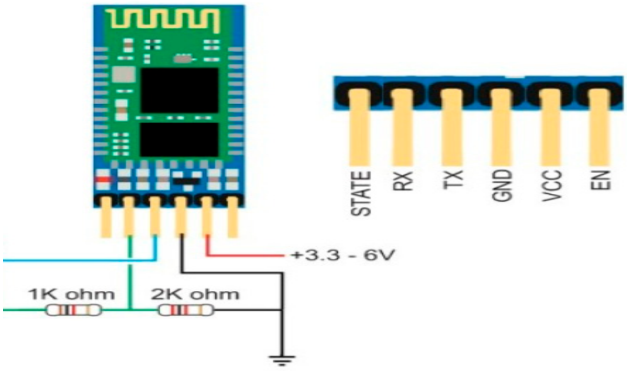

Figure S2. Bluetooth module connection with Arduino.
